# P-2060. Sociodemographic Disparities and Their Possible Impact on the Epidemiology of Pediatric Community Acquired Bacteremia

**DOI:** 10.1093/ofid/ofaf695.2224

**Published:** 2026-01-11

**Authors:** Michal Barzel, Eli Somekh, Alexandra Gleizer, Judith Shindler, Orna Schwartz, Maya Heled-Akiva, Diana Tasher

**Affiliations:** Department of Pediatrics, Mayanei Hayeshua, Bnei-Brak, Israel, Bnei Brak, HaMerkaz, Israel; Department of Pediatrics, Mayanei Hayeshua, Bnei-Brak, Israel, Bnei Brak, HaMerkaz, Israel; Maccabi Health Services, Israel, tel aviv, HaMerkaz, Israel; Clinical Microbiology Laboratory, Mayanei Hayeshua, Bnei-Brak, Israel, Bnei Brak, HaMerkaz, Israel; Clinical Microbiology Laboratory, The Edith Wolfson Medical Center, Holon, Israel,, Holon, HaMerkaz, Israel; Wolfson Medical Center, Holon, HaMerkaz, Israel; Pediatric Infectious Diseases Unit, Edith Wolfson Medical Center, Holon /Sackler School of Medicine, Tel-Aviv University, Holon, Tel Aviv, Israel

## Abstract

**Background:**

Data on the influence of sociodemographic characteristics on the epidemiology of community-acquired bacteremia in vaccinated populations remains limited. We aimed to examine the association between sociodemographic characteristics and the epidemiology of pediatric community acquired bacteremia.

**Figure:**
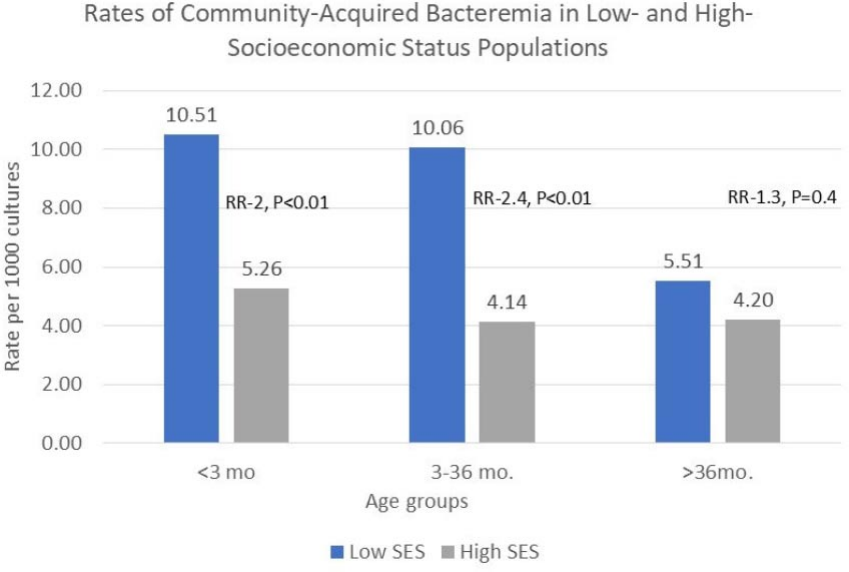
Rates of Community Acquired Bacteremia in Low and High Socioeconomic Status Populations

**Methods:**

This retrospective study was conducted in two hospitals serving populations with significant socioeconomic disparities (low socioeconomic status [SES] rank 2/10 vs. high SES: rank 7/10, based on the Central Bureau of Statistics' scale). We included all cases of community-acquired bacteremia in patients< 18 years between 2016-2023. Bacteremia rates were calculated per 1,000 blood cultures drawn. Rates of true bacteremia and the distribution of isolates were compared between hospitals. *S.pneumoniae* isolates were further analyzed to determine serotype coverage by the Prevenar 13 and Prevenar 20 vaccines.

**Figure:**
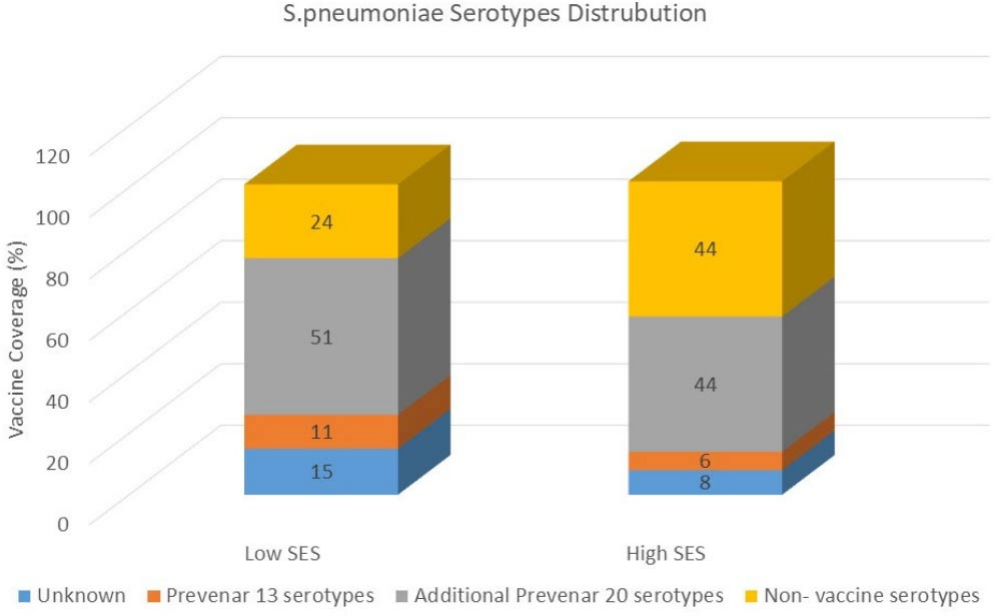

**Results:**

We identified 298 cases of community-acquired bacteremia. Bacteremia rates were higher in low- vs. high-SES children < 3 years (10.5 vs. 5.2 cases/1,000 cultures; RR -2, P < 0.01 and 10.06 vs. 4.1 per 1,000 cultures; RR-2.4, P < 0.01, in those aged < 3 months and 3–36 months, respectively). *S.pneumoniae* was the leading isolate among children aged 3–36 months (n=55, 44.3% in low vs. n=36, 22.3% in high-SES populations). In the low-SES population*, S.pyogenes* (10.4%) and *N.meningitidis* (7.0%) were the next most common isolates, while *K.kingae* (16.0%) and *M.catarrhalis* (11.7%) followed in the high-SES population. Non-Prevnar 13 *S.pneumoniae* serotypes accounted for 94.4% of cases in high-SES and 89% in low-SES populations (P = 0.4). Prevnar 20 covered isolates accounted for 50.9% in low-SES and 38.8% in high-SES populations (P = 0.2).

**Conclusion:**

Low-SES children < 3 years had more than twice the risk of bacteremia compared to high-SES children, with higher rates of *S.pyogenes* and *N.meningitidis*. Non–Prevnar 13 *S. pneumoniae* serotypes accounted for the majority of cases in both populations, with a substantial proportion covered by the recently introduced Prevnar 20 vaccine, highlighting its potential to reduce the burden of invasive pneumococcal disease.

**Disclosures:**

All Authors: No reported disclosures

